# Safety Challenges and Application Strategies for the Use of Dendrimers in Medicine

**DOI:** 10.3390/pharmaceutics14061292

**Published:** 2022-06-17

**Authors:** Xiang Li, Abid Naeem, Shanghua Xiao, Lei Hu, Jing Zhang, Qin Zheng

**Affiliations:** 1 National Pharmaceutical Engineering Center for Solid Preparation in Chinese Herbal Medicine, Jiangxi University of Chinese Medicine, Nanchang 330006, China; xiang.li@jxutcm.edu.cn (X.L.); hulei@jxutcm.edu.cn (L.H.); 2 Key Laboratory of Modern Preparation of TCM, Ministry of Education, Jiangxi University of Chinese Medicine, Nanchang 330004, China; 5862018301@jxutcm.edu.cn (A.N.); xiaoshanghua@jxutcm.edu.cn (S.X.)

**Keywords:** dendrimer, exposure routes, distribution, toxicity, safety, surface functionalization

## Abstract

Dendrimers are used for a variety of applications in medicine but, due to their host–guest and entrapment characteristics, are particularly used for the delivery of genes and drugs. However, dendrimers are intrinsically toxic, thus creating a major limitation for their use in biological systems. To reduce such toxicity, biocompatible dendrimers have been designed and synthesized, and surface engineering has been used to create advantageous changes at the periphery of dendrimers. Although dendrimers have been reviewed previously in the literature, there has yet to be a systematic and comprehensive review of the harmful effects of dendrimers. In this review, we describe the routes of dendrimer exposure and their distribution in vivo. Then, we discuss the toxicity of dendrimers at the organ, cellular, and sub-cellular levels. In this review, we also describe how technology can be used to reduce dendrimer toxicity, by changing their size and surface functionalization, how dendrimers can be combined with other materials to generate a composite formulation, and how dendrimers can be used for the diagnosis of disease. Finally, we discuss future challenges, developments, and research directions in developing biocompatible and safe dendrimers for medical purposes.

## 1. Introduction

Dendrimers are hyperbranched and monodispersible macromolecules that contain symmetric branches encircling a small molecule or linear polymeric core and are characterized by a cavity structure, functional surface groups, and controllable physicochemical properties. Due to their unique properties, dendrimers have been widely used in the medical health industry [1,2]. However, comprehensive toxicity studies on dendrimers have been relatively one-sided and incomplete. Herein, we discuss the categories of dendrimers and their key properties related to toxicity. We also provide an in-depth discussion of these issues, including the multifaceted toxicological effects and mechanisms of dendrimers. Finally, we provide detailed insights into the applications of modified dendrimers in theranostics.

## 2. Classification, Types, and Properties of Dendrimers

### 2.1. Classification of Dendrimers

Dendrimers are classified according to the number of generations, as determined by the degree of branching. A linear molecule can be added to the reaction site of the initial core molecule; thus creating a “stem” of a tree, to which two additional linear-like structures are attached via the ends of the “stem” to form a “Y”-shaped structure. Repeating this pattern, that is, adding a “Y”-shaped structure at the free end of the “Y”, will generate more advanced structural morphology. When this repeating pattern occurs many times, a similar structure is generated that resembles a three-dimensional sphere. The number of “Y”-shaped structures in the structure increases algebraically [3,4]; these morphological differences are referred to by the number of generations (G0-Gn) [5].

### 2.2. Types of Dendrimers

#### 2.2.1. Monomeric Dendrimers

**Figure 1:**
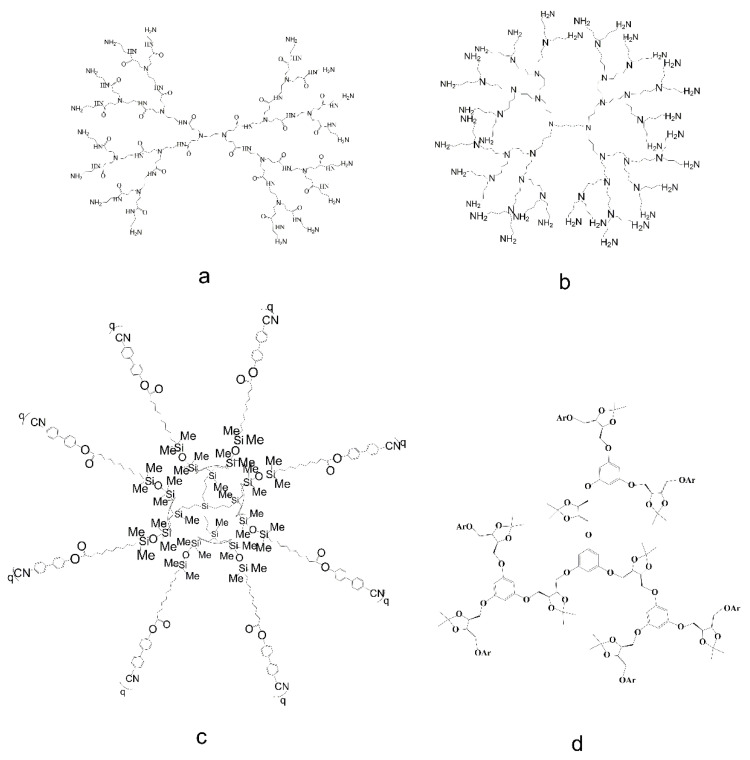
The structures of four major monomeric dendrimers: (**a**) Poly (amidoamine); (**b**) Poly (propylene imine); (**c**) Carbosilane; (**d**) Chiral dendrimers.

Dendrimers are synthesized in a stepwise manner from branching monomer units. By employing a variety of building/branching units, as well as functional groups on the surface, we can gain precise control of shape, molecular size, density, dimension, flexibility, polarity, and solubility. Furthermore, dendrimers can be constructed using polymers and small organic molecules as structural components, thus yielding specific chemical and physical properties. The structures of the four major monomeric dendrimers are as follows (Figure 1) [6,7,8,9]:

##### Poly (Amidoamine) (PAMAM) Dendrimers

PAMAM dendrimers are composed of central ethylenediamine functional groups, polyamide branches, and terminal multifunctional groups; these are now in their tenth generation. It is worth noting that cationic and neutral PAMAM dendrimers are generated as integer generations of tree-like macromolecules (G1 to G10), while anionic PAMAM dendrimers can be produced in half generations (G1.5 to G9.5) [6].

##### Poly (Propylene Imine) (PPI) Dendrimers

These dendritic macromolecules possess 1,4-diaminobutane as the core and tertiary amine spacers as the branch molecules [10]. The outer part is the primary amine, and the inner part is the tertiary amine group; thus, these are often referred to as butane (DAB) or polypropylene amine dendrimers [7,11]. A recent study found that an unmodified PPI (G4) dendrimer induced apoptosis in 60% of vascular endothelial cells, due to the presence of terminal positive groups, while a glycosyl-modified PPI (G4) dendrimer exhibited lower levels of endothelial cytotoxicity [7]. These studies demonstrate the importance of surface modifications on dendrimers.

##### Liquid Crystalline (LC) Dendrimers

LC dendrimers are typically prepared from liquid crystals that contain carbosilane dendrimers [12]. Molecules that are generally discoid or rod-shaped play an important role in the formation of liquid crystal phases. In addition, LCs contain poly (propylene imide) [13,14], poly (amidoamine) [15], stilbene [16], and ferrocene structures [8,17]. Dendritic cores and functionalized terminals often present with microphase separation, due to incompatible chemical properties. Therefore, Donnio et al. regarded these as block molecules similar to block polymers [18]. Previous researchers investigated LC dendrimers of different generations and found that compared with other lower generations, fifth generation LC dendrimers could not form cylindrical morphologies, due to their lack of a mesocrystalline structure; however, LC dendrimers of lower generations could form a cylindrical morphology, due to their hexagonal intermediate nucleus [19]. It is worth noting that with the help of LC dendrimers, guest molecules can be better aligned and, remarkably, display the physical properties of guest molecules, when solvating guest materials, such as carbon nanotubes and nanoscale catalysts [20,21]. Lu et al. reported the preparation of triazine-based LC dendrimers, and their thermal and electrochemical stability makes them useful in opto- and electro-applications [21,22,23]. A polymer network based on LC dendrimers via the crosslinking of two reactive dendritic entities (codendrimer I and codendrimer II) was constructed. It was varied by adjusting several structural parameters and could be applied in potential fields, such as sensors, catalysis, and drug delivery [24].

##### Chiral Dendrimers

Chiral dendrimers possess a unique stereochemical structure, which can be used for molecular recognition and asymmetric catalysis [3,12]. These dendrimers are composed of units with different structures but with similar chemical properties; in other words, these dendrimers possess chirality [11]. Generally, chiral dendrimers can be artificially divided into three categories based on the chirality of the construction unit (the nuclear unit is a chiral group, the construction unit is a chiral group, and the nuclear unit and the construction unit are both chiral groups). Chiral dendrimers were first described in 1993, when Seebach et al. successfully prepared chiral cores for dendrimers by chemical methods [25]. In 2004, Ghorai et al. were the first to introduce anthracene-capped chiral dendrimers with 1,3,4-trisubstituted aromatics as the core and carbohydrates as the surface group; these dendrimers were promising for application in drug delivery and as light treatment materials [26]. Recently, Malinga-Drozd [27] et al. transformed G2 and G3 PAMAM into N-(2,3-dihydroxy) propyl derivatives by adding purified enantiomers R-and S-glycidyl. The obtained PAMAM-based chiral dendrimers were incubated with HaCaT and scc-15 cells. These chiral dendrimers aggregated in cells in a time-dependent manner and with good biocompatibility, which makes them an excellent carrier for drug delivery. Another in vitro study was conducted on the G3 PAMAM-based chiral dendrimer, and this showed that this chiral dendrimer also has great potential for combating glioblastoma [28].

##### Cationic Phosphorus (CPD) Dendrimer

This is a class of dendrimers with a positive charge on the surface and phosphorus in the structure, which specifically means that each branch node contains a phosphorus atom [29]. They are characterized by high potential medical value, and often used in the treatment of HIV [30], prions [31], and cancer [32]. Recently, Chen et al. constructed cationic phosphorus containing dendrimers with different generations and different cyclic amine ends, to optimize gene delivery for cancer gene therapy applications [33].

#### 2.2.2. Multicomponent Dendrimers

##### Core–Shell (TECTO) Dendrimers

**Figure 2:**
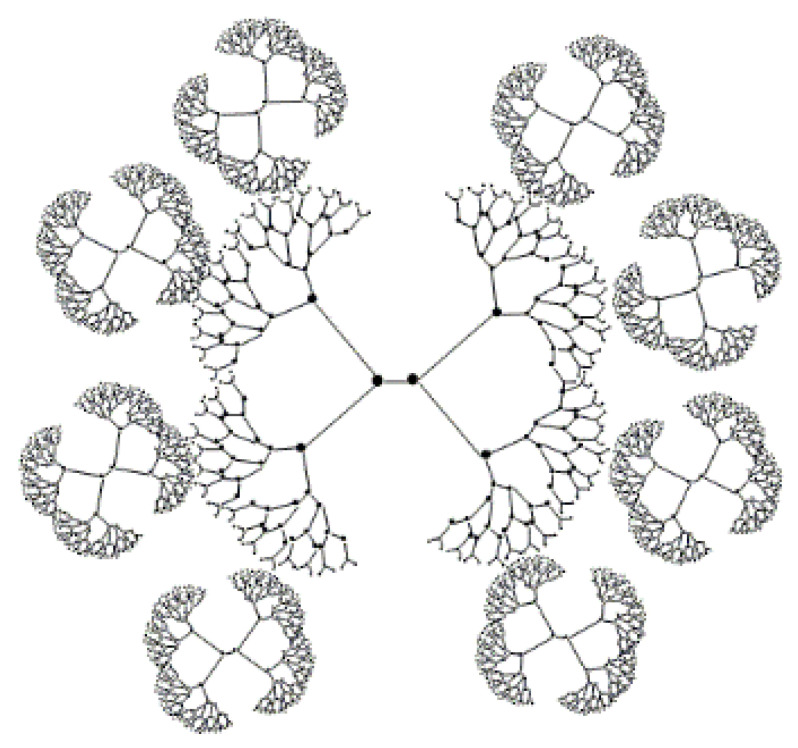
Schematic representation of a PAMAM core–shell TECTO dendrimer.

Core–shell dendrimers are a distinct class of dendrimers with a peculiar structure (Figure 2). Dendrimers form the core of this structure, while the outer shell is composed of covalent dendrimers. The outer shell wraps around the core and forms core–shell dendrimers. The generations of internal and external dendrimers that make up the structure of the TECTO dendrimer are different. A high generation dendrimer is used as the core, while a low generation dendrimer is used as the shell [34,35]. Uppuluri et al. connected a high generation PAMAM (G5, G6, G7) core with a low generation PAMAM (G3, G5) shell using amide bonds, to form a new type of dendrimer that became known as a core–shell dendrimer [36]. In 2012, Schilrreff et al. synthesized TECTO-dendrimers using PAMAM G5 as the core and PAMAM G2.5 as the shell; the experimental results showed that these dendrimers had good anti-melanoma activity at safe concentrations [37]. The recent research on using Tecto dendrimers as a drug delivery system is also very interesting. Researchers prepared the PAMAM G3-G0 drug delivery system for delivering antitumor drugs. The results show that the PAMAM G3 core-G0 shell-nimesulide can effectively penetrate cells at micro molar concentration and have a selective anticancer activity against human squamous carcinoma and glioma cells, by inhibiting cell proliferation [38].

##### Peptide Dendrimers

**Figure 3:**
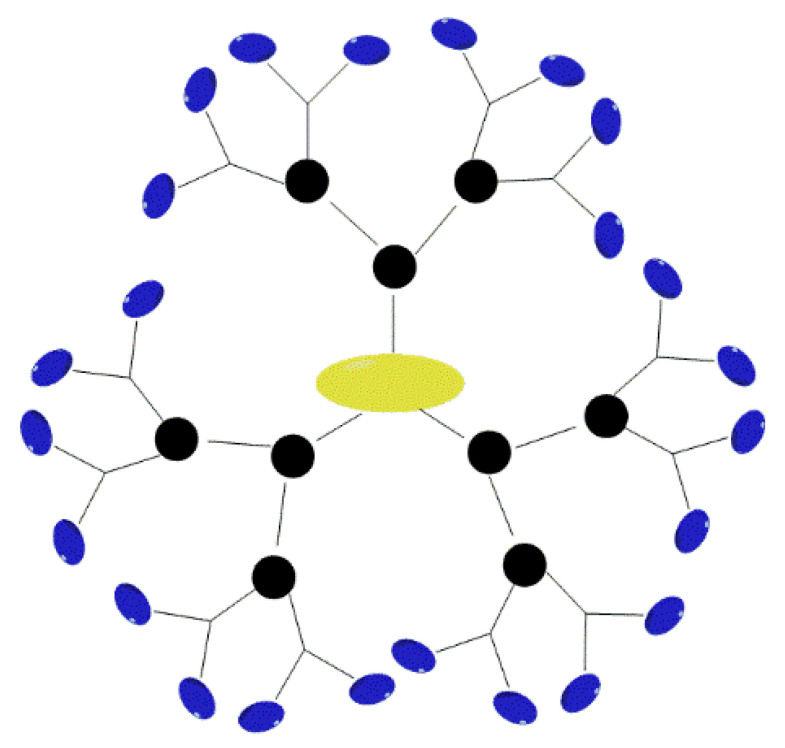
Typical structure of a peptide dendrimer.

Peptide dendrimers feature peptide bonds on the surface or in the dendritic structure composed of amino acids (Figure 3) [1,39] and can be divided into three categories. The first type are dendritic macromolecules whose entire structure is made up of amino acids. The second type are dendritic macromolecules whose core and branching units are composed of amino acids and whose terminal groups are composed of non-peptide branching units. The third type are grafted peptide dendrimers consisting only of peptides as outer surface functional group [12].

##### Glyco-Dendrimers

**Figure 4:**
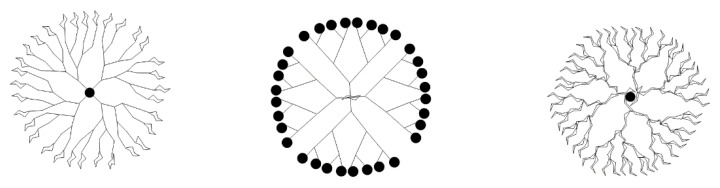
Different types of glyco-dendrimers.

As the name suggests, glyco-dendrimers contain carbohydrate [12]. The structure of glycol-dendrimers often contains glucose, mannose, galactose, or disaccharide. There are three types of dendrimers: the first type are sugar-covered dendrimers, the second type are dendrimers with sugar as the core, and the third type are sugar-based dendrimers (Figure 4) [40,41].

### 2.3. Properties of Dendrimers

#### 2.3.1. Nanoscale and Monodispersity

Dendrimers are characterized by a specific molecular weight, molecular scale, and a well-defined structure. Furthermore, their molecular volume and morphology can be precisely controlled. These are similar in shape and size to many proteins and cells. The molecular size of dendrimers increases with the generation, ranging from a few nanometers to tens of nanometers in diameter. The low-generation dendrimers exhibit an open morphology, while the high-generation dendrimers exhibit a regular, spherical morphology [2].

#### 2.3.2. Biocompatibility

Biocompatibility has been defined as “the ability of a material to react appropriately with a host in a specific setting” [42]. To gain an appropriate understanding of how a biomaterial will affect a host, it is first necessary to determine both its toxic and beneficial effects on the physiological environment [40]. Dendrimers, such as PAMAM and PPI, are the most frequently used carriers for delivering drugs. However, dendrimers containing terminal amine groups, such as PAMAM and PPI, may exhibit toxic effects. Of the two types of dendrimers, PAMAM dendrimers exhibit potential toxicity, based on their generation and concentration. As the number of generations increases, so does the concentration of the surface amine group, thus increasing the level of toxicity [43]. Furthermore, positive charges on the amine groups render the dendrimers capable of interacting with negatively charged proteins in a cell, thus causing lysis of the cell membrane. Dendrimers that contain neutral or anionic terminal surface groups are unlikely to cause cell toxicity and have been demonstrated to be non-toxic. As a result, the cationic groups on the surface of PAMAM dendrimers and PPI dendrimers can be changed into neutral or anionic groups, to reduce their toxic properties. The cationic dendrimers PAMAM and PPI have been modified extensively, in an attempt to reduce their toxic properties [44]. Dendrimer modification strategies such as glycolation [45,46], folate conjugation [47], acylation [48,49], and PEGylation [50] have been demonstrated to exhibit less toxicity in vitro or in vivo. Moreover, the toxicity of dendrimers can also be reduced by adding compounds such as peptides [51], carbohydrates [52], and amino acids (such as glycine and phenylalanine) [53] that reduce positive charges on the surface of the dendrimers. In addition, dendrimer toxicity can be significantly reduced by combining/conjugating DNA and drugs with dendrimers, and forming complexes by coating their surface with negative charges via DNA that is attracted to its positive charges [54]. The other alternative is to synthesize non-toxic biodegradable dendrimers, such as polyester and polyetherdendrimers [12,19], which are both biodegradable.

#### 2.3.3. Special Structures

Dendrimers are a form of macromolecular polymer with a tree-like structure and consist of three parts: a core, a branch unit, and a terminal group. In the low generations, these take the form of an open structure; while, in higher generations, they take on the form of three-dimensional spheres that are tight outside and loose inside. The unique function of these dendrimers is determined by their peculiar morphological structure. For example, these can be highly efficient drug delivery vehicles [54,55,56]. Drugs can be coated into the interior of these dendrimers or modified onto the terminal groups, to play a pharmacodynamic role. 

#### 2.3.4. Polyvalent Surfaces

The surface groups of dendrimers are multivalent, which may be positive, negative, or neutral, and can react with multiple receptors. Surface groups with different valence states can relate to a variety of molecules with different functions, such as drug-dendrimers, antibody-dendrimers, bioactive molecule-dendrimer conjugates, and diagnostic agent-dendrimers [3,57,58]. It is worth noting that positive terminal groups may lead to serious cytotoxicity; however, as described above, such effects can be reduced by surface modification, valence adjustment, and other means.

## 3. Dendrimer Toxicity

As potential nanocarriers, dendrimers have contributed to significant breakthroughs in the field of medicine; however, toxicity research is of great significance for the safe application of dendrimers. The toxicity and other properties of these special compounds are directly related to their structure. Due to their nanoscale size, dendrimers may interact specifically with other nanoscale cellular components, such as the plasma membrane, organelles (endosomes, mitochondria, nuclei), proteins (enzymes), heavy metals, ions, vitamins, and nucleic acid (NA) [12,59]; these events can then lead to membrane rupture [60], the production of ROS, and the release of cytokines, ultimately causing cell damage and cell death [61].

### 3.1. Toxicity-Related Physicochemical Properties

#### 3.1.1. Size and Generation

The toxicity of dendrimers is dependent on their generation and size. The cytotoxicity of high generation cationic dendrimers is generally higher than that of low generation dendrimers; ascribed to the increased number of positive charges [62,63,64,65]. To a large extent, only high-dose or high-generation cationic dendrimers exhibit a certain toxicity in vivo, and this toxicity can be reduced by the modification of the dendrimers [66]. It has been shown that PAMAM G6-NH_2_ dendrimers can cause total hemolysis within 2 h and that G4-NH_2_ and G5-NH_2_ dendrimers cause 100% hemolysis within 4 h, while PAMAM G3 dendrimers reach a maximum of 80% hemolysis after 24 h [67]. Similarly, aggregation was previously detected in platelet-rich plasma when treated with G4, G5, and G6 amino-terminal dendrimers (except for PAMAM G3-NH_2_ dendrimers) [68]. In addition, dendrimers with terminal hydroxyl and terminal carboxyl groups do not cause the aggregation of human platelets in vitro, regardless of their size. Some authors have suggested that the larger the generation of cationic dendrimers, the higher the cation charge, and indirectly leading to greater levels of hemolysis [69]. The toxicity investigation of PAMAM dendrimers G3.0, 3.5, 4.0, 4.5, and 5.0 and PPI dendrimers G3.0, 4.0, and 5.0 in zebrafish embryos and human cancer cell lines showed that the IC_50_ values of the dendrimers were different for different cell lines. The IC_50_ values of PAMAMs were above 402 μM and below 13.24 μM for HepG2 and DU145, respectively. The IC_50_ values of PPIs were blow 240 μM and 12.84 μM for HepG2 and DU145, respectively. Although the toxicity of HepG2 and DU145 cell lines increased with the increasing number of cationic PAMAM and PPI dendrimers, low-generation dendrimers were clearly more toxic to embryos. This might be explained by the negatively charged chorion of Zebrafish embryos, which can interfere with the interaction of high-generation cationic dendrimers; while low-generation cationic dendrimers are not easily interfered with and show stronger toxicity [70].

#### 3.1.2. Charge

Interestingly, in vivo studies of the embryonic zebrafish model have shown that the surface charge properties of dendrimers is a more important factor in the cytotoxicity of dendrimers than either generation or size [71]. When dendrimers were applied as nanocarriers, positively charged dendrimer cores exhibited significant cytotoxicity and severe phenotypic changes, compared with negatively charged dendrimer cores [72].

The toxicity of dendrimers has been shown to mainly depend on the surface charge properties (cationic, anion, and neutral). Cationic dendrimers generally exhibit increased levels of toxicity, although low concentrations of these substances can be considered safe [69]. For example, amine terminated PAMAM dendrimers (G4 and G7) are safe only at doses ≤10 mg/kg, while carboxyl (G3.5 and G6.5) and hydroxyl (G4 and G7) terminated dendrimers are safe at a dose that is 50-fold higher in caesarean derived-1 mice [62]. Neutral (CSi-PEO) dendrimers (concentrations < 1 mg/mL) exhibited no hematologic toxicity or cytotoxicity [73]. Therefore, G7-NH_2_ PAMAM dendrimers with a large positive zeta potential, and G7-OH with a slightly lower positive zeta potential, showed acute signs of toxicity at higher doses; however, other low-potential dendrimers (G3.5-COOH, G4-NH_2_, G4-OH, and G6.5-COOH) did not show any significant signs of toxicity [74]. When incubated with fresh rat blood cells, all the amine-terminated PAMAM dendrimes were found to be hemolytic, dependent on the concentration and generation, and changed the morphology of red cells even at 10 μg/mL (non-hemolytic concentration). Anionic dendrimers caused no changes in red cell morphology until 2.0 mg/mL [73]. Agashe et al. investigated the hematological toxicity of G5.0 PPI dendrimers. They observed a significant decrease in red blood corpuscles count, by 38%, which may have been attributed to the overall cationic charge [53]. 

This result may have been related to the zeta potential of the dendrimers. Dendrimers with amino terminal groups had a positive zeta potential, while carboxylic acid terminated dendrimers had a negative zeta potential. For example, dendrimers with a hydroxyl (OH) terminal had an almost neutral zeta potential (G2-OH), while G7-OH dendrimers had a charge of −4.8 mV and 2.8 mV, respectively [75]. The polycationic nature of dendrimers makes them interact more easily with negatively charged cell membranes, then with other cellular components, causing apoptosis and other toxic mechanisms [69]. 

#### 3.1.3. Concentration

The toxic concentrations of cations can be expressed as the maximum tolerance value; a value greater than the maximum tolerance dose (MTD) shows concentration-dependent toxicity, while anions and neutrals rarely express cytotoxicity over a large concentration range (except for extremely high concentrations), both in vivo and in vitro. For the in vivo toxicity of PAMAM dendrimers in the mouse gut, the MTD of cationic dendrimers was in a range of 30–200 mg/kg, while the MTD of anionic dendrimers was as high as 500 mg/kg (below G7) [74]. Of course, in addition to MTD, hemolytic concentration is also an intuitive evaluation index of toxicity. High concentrations of high generation cation dendrimers (at 90 nM, G6) express the highest levels of hemolytic toxicity and cytotoxicity. Low concentrations of high generation toxicity are also strong; however, below a certain value (below 0.009 nM, G6), high generation cations express no toxicity [67]. Roberts et al. previously evaluated in vivo toxicity, by studying behavioral abnormalities and body weight changes within 6 months after the injection of cationic PAMAM G3, G5, and G7. These authors found that a safe dose of cationic PAMAM caused no significant behavioral abnormalities or weight loss in vivo [76]. When treated with G7-NH_2_ dendrimers, human umbilical vein endothelial cells showed high levels of toxicity at concentrations of 5 to 100 μg/mL, while G2-NH_2_ dendrimers only showed weak cytotoxicity. In contrast, neither neutral nor anionic PAMAM dendrimers (G2-OH, G7-OH, G1.5-COOH, and G7.5-COOH) showed significant toxicity at concentrations of 1 to 100 μg/mL [75]. The calculated IC_50_ of the carbosilane glucose glycodendrimers in three different grey hamster cell lines, B14, BRL 3A, and NRK 52E, was in the mM range, while the IC_50_/EC_50_ of glycodendrimers G3 in embryos was greater than 100 µM, indicating that the carbosilane glucose glycodendrimers have a very low cytotoxicity and toxicity in vivo [77].

### 3.2. Toxicity to Cells

It is important to consider the toxicity mechanisms of dendrimers and the toxicity in normal or cancer cell lines from humans and animal models. In vitro cell line research plays an important role in the characterization of dendrimer toxicity and provides a basis for the correlation of in vivo research. Normal and cancer cell lines from various animal and human sources are used to characterize biological activity. 

Table 1 summarizes the cytotoxicity of dendrimers to human- and animal-derived cell lines and the mechanisms involved. 

#### 3.2.1. Direct Interaction with Membrane

The lipid bilayer, mainly composed of phospholipids and cholesterol and embedded with proteins, forms an overall negative charge in the plasma membrane. Cationic dendrimers interact with the negative charge of the plasma membrane via electrostatic interaction, thus resulting in the formation of pores (nanopores); this leads to a further increase in plasma membrane permeability and, thus, cell lysis [59]. In the systemic circulation, cationic dendrimers can interact with blood components, destroy the stability of the cell membrane, and lead to hemolytic toxicity.

Dendrimers of different generations can interact with the membranes of blood cells and cause hemolytic toxicity. Cationic dendrimers induce the hemolysis of RBCs and platelet aggregation by disrupting the integrity of the platelets and RBC membranes. A study of the interaction between cationic carbosilane dendrimers and erythrocytes previously found that carbosilane dendrimers could increase the internal fluidity of hydrophobicity and induce hemolysis without changing the morphology of RBCs [102]. Different generations of PPI dendrimers were previously used to study hemolytic toxicity; the results showed that hemolytic toxicity increased with the number of generations. For example, a PPI G5 dendrimer significantly changed the shape and hemolysis rate of RBCs, exhibiting a hemolysis rate of 6.39%; however, the PPI G5 dendrimer can still be used for drug delivery [4]. In addition, cationic dendrimers can induce leukocyte coagulation via a mechanism similar to Adriamycin and thus aggravate the procoagulant activity induced by endotoxin [93]. 

#### 3.2.2. Apoptosis and Autophagy

First, cationic dendrimers are internalized into cells and then released from endosomes or lysosomes into the cytoplasm [103]. Then, dendrimers are localized to the mitochondria and nuclei [104,105]. Studies have shown that PAMAM dendrimers can reduce the transmembrane potential and prevent calcium ions from flowing into mitochondria, so as to regulate mitochondrial function [106]. Oxidative stress in the mitochondria is closely related to apoptosis. For example, the apoptosis in human macrophages caused by PPI dendrimers [107], and in mouse embryonic hippocampal cells and neuroblastoma cells caused by CPD dendrimers [108], is closely related to the production of ROS. Dendrimers can induce apoptosis by affecting other signaling pathways related to the regulation of cell physiological activities. In a previous study involving the HER2-positive human breast cancer cell, PAMAMs were able to induce apoptosis in numerous cancer cells by enhancing the expression of c-Jun N-terminal kinase (JNK) 1/2/3, while blocking the expression of extracellular-regulated kinase 1/2 (ERK 1/2) [87]. Furthermore, PAMAMs can induce the hyperphosphorylation of ERK1/2 and p38 mitogen-activated protein kinase (p38 MAPK) and apoptosis; this may occur through an epidermal growth factor receptor (EGFR) -independent and oxidative-stress dependent mechanism [109].

Dendrimers can exert pro-inflammatory and pro-apoptotic effect via cytokines; therefore, cytokines can play an important role in the toxicity of a dendrimer. In mouse macrophages, Herzog et al. observed the cytotoxicity of PAMAM dendrimers (G4, G5, and G6) and found that the mechanism of toxicity was related to the production of ROS in cells. This led to the cascade secretion of pro-inflammatory markers (cytokines), eventually leading to cell death [110]. In another study, involving the response of macrophage cells to PAMAM dendrimers, IL-6, MIP-2, and TNF-α were found to be secreted by J774A.1 cells, and the level of secretion increased concurrently with the generation of the PAMAM dendrimer. Thus, generation-dependent release of ROS appeared to be correlated with the increase in the number of surface primary amino groups [91]. 

Dendrimers can interact with nucleic acids, leading to a functional change that leads to cell cycle propagation and apoptosis. Exposure to low doses (no cytotoxicity) of cationic PAMAM-NH_2_ dendrimer resulted in NF-κB-dependent cell cycle arrest in primary human bronchial epithelial cells, but not in lung cancer cells [79]. Plasmid relaxation assays failed to detect CPD G3- and G4-induced apoptosis in A549 cells. However, comet assays showed that dendrimers cross-linked with DNA, thus affecting the DNA concentration. This interfered with the signal transmission between proteins and DNA, blocking DNA strand replication; thereby leading to cell cycle arrest in certain cells, followed eventually by apoptosis [98]. CPD G2 and G3 also induced the death of mouse embryonic hippocampal cells by producing ROS, reducing cell activity, affecting the cell cycle, and damaging DNA [108]. In addition, apoptosis and necrosis may sometimes occur simultaneously. DNA cleavage and apoptotic ladders were detected in RAW 264.7 murine macrophage-like cells when treated with cationic PAMAM and DAB dendrimers, thus indicating the apoptotic and necrotic effects of PAMAM and DAB dendrimers [111]. 

Autophagy is also a major pathway by which dendrimers can cause toxicity; this pathway also has a certain relationship with apoptosis and ROS. PAMAM dendrimers damage mitochondria and induce apoptosis by inhibiting the serine threonine kinase/ mammalian target of rapamycin (Akt/mTOR) pathway and by activating the ERK1/2 signaling pathway in human hepatocytes [85]. In a previous study of neurotoxicity in human glioblastoma cell lines, Wang et al. found that autophagy was initiated by the Akt/mTOR pathway and partially mediated by ROS. Furthermore, these authors also found that the inhibition of autophagy significantly reversed the neural cell death caused by PAMAM dendrimers [83]. The cytotoxicity of PAMAM G5 dendrimers in a HepG2 cell could also be inhibited by autophagy inhibitors, thus indicating the important role of autophagy inhibition in PAMAM-induced cytotoxicity [85]. Interestingly, the specific induction of autophagy in A549 cells, by deregulating the Akt-TSC2-mTOR signaling pathway with PAMAM dendrimers G3, appeared to have no relationship with apoptosis [94].

#### 3.2.3. Interaction with Other Cellular Components

The interaction between dendrimers and other cellular components can influence physiological activities in a beneficial manner but can also bring about certain disadvantages. Most blood protein is negatively charged on the surface; thus, cationic dendrimers may interact with the active sites of these proteins, resulting in hemolytic toxicity, with non-specific clotting and the degradation of clots simultaneously [112]. It was also reported that amine-terminated dendrimers can non-specifically initiate the formation of fibrinolytic fibrin fibers at low concentrations of thrombin [113]. Other researchers investigated the effect of the surface charge of PAMAM dendrimers on enzyme activity; these researchers showed that PAMAM dendrimers with positive and neutral charges could both inhibit the activity of pepsin [114]. Interestingly, Thomas et al. tested the effect of cationic PAMAM G5 dendrimers on the lysosomal pH of KB cells and found that the rapid accumulation of cationic molecules in lysosomes led to changes in pH and apoptosis [84]. In addition, dendrimers have also been shown to complex with some metal ions, such as iron and zinc, and affect the transport function of hemoglobin, and the functionality of catalase in the kidneys, respectively [60].

### 3.3. Toxicity to Tissues

Although there are abundant studies on the toxicity of dendrimers at the cellular level (in vitro), there are very few studies on dendrimers in vivo; this is due to the complex physiological activities and components in vivo. However, cytotoxicity cannot fully represent the toxicity of dendrimers within the body. When considering the safety of drug use, it is important to study toxicity in the body and not just in vitro. To study the toxicity of dendrimers in different parts of the human body, we analyzed the toxicity of dendrimers in different animal and isolated human tissues in vivo (Table 2).

#### 3.3.1. The Hematological System

Dendrimers promote sustained systemic distribution by remaining in the blood and by remaining in contact with blood flow components for long periods of time [120]. However, when cationic dendrimers enter the blood, they interact with cells (RBCs, white blood cells, and platelets) and proteins (e.g., hemoglobin) in the blood, thus resulting in hemolytic toxicity and changes in hematological parameters.

By disrupting the integrity of the plasma membranes of platelets rather than a specific signal, cationic PAMAM dendrimers G4, G5, and G6 were shown to result in the aggregation of human platelets, as determined by particle size and surface charge analysis [68]. However, triazine dendrimers exhibited far less aggregation and thrombogenic properties in the blood; this was due to the lower density of surface amines [61]. It was also reported that cationic PAMAM G7 dendrimers initiated both the clotting and degradation of clots simultaneously, inducing disseminated intravascular coagulation-like complications after intravenous administration to mice; these complications could be fatal at doses >10 mg/kg [62]. The inhibition of phosphoinositide 3 kinase (PI3K) by dendrimers led to the amplification of endotoxin-induced leukocyte PCA; this is the key factor underlying DIC [121].

#### 3.3.2. The Immune System

Although dendrimer nanocarriers are used to avoid immunotoxicity, they may also cause immunotoxicity in the body [122]. Roberts et al. studied the immunogenicity of the cationic PAMAM dendrimers G3, G5, and G7 with an amino surface in rabbits by immunoprecipitation and Ouchterlony double-diffusion assays, but could not detect an immunogenic response [76]. In another study, Agashe et al. investigated the immunogenicity of PPI dendrimers in Balb/c mice by ELISA, but could not detect an antibody titer (IgG) at 450 nm at any concentration tested; this indicated that dendrimers were unable to induce a humoral immune response under these particular experimental conditions. This also suggested that PPI dendrimers are unable to induce immunogenicity [53]. The influence of PAMAM G5 dendrimers with amine, hydroxyl, or carboxyl on the secondary structure and conformation, as well as on the complement of immune molecule γ-globulin, was also reported. In this research, dendrimers were found to affect the intensity of the peaks, the microenvironment around the tryptophan residues, and the conformational change of γ-globulin, as determined by ultraviolet-visible, fluorescence, and circular dichroism spectroscopy. These dendrimers also reduced the production of complement C3a, as determined by ELISA [123].

#### 3.3.3. The Nervous System

Drug delivery in the central nervous system is challenging because the blood–brain barrier (BBB) blocks most drug delivery systems from entering the brain. In addition, the toxicity of drug delivery carriers to the central nervous system is also a major problem [124]. Dendrimers can deliver drugs/genes to treat brain diseases or nervous system diseases via the BBB, but their potential for neurotoxicity cannot be ignored [125]. Similarly, due to their structural characteristics, dendrimers have been proven to induce numerous neurotoxicological reactions in organisms.

PPI dendrimer-induced autophagy can produce adverse effects such as ataxia (difficulty moving smoothly and accurately) in the nervous system of rats, and their neurotoxicity accumulates over time [115]. A previous study of human neural progenitor cells demonstrated the inhibitory effect of PAMAM-NH_2_ dendrimers on neuronal proliferation and migration [81]. Another study relating to neurotoxic effects on human neural progenitor cells showed that dendrimers could affect oxidative stress, apoptosis/DNA damage, and the development of neural cells, and that PAMAM dendrimers may have adverse effects on neuronal differentiation that are surface charge-dependent [126]. PAMAM dendrimers were also shown to upregulate inflammatory receptors on microglia in vitro and cause neuroinflammatory reactions [127]. The proliferation and differentiation of neurons and neuroinflammation may also produce potential effects in vivo.

#### 3.3.4. Organ Toxicity

PAMAM dendrimers can be biologically distributed in the heart, lungs, liver, blood, urine, stomach, and small and large intestine following oral administration [128]. The slow clearance and tissue accumulation of potential ROS-producing dendrimers, as well as the widespread presence of phagocytes, renders organs such as the liver and spleen the main targets of dendrimer toxicity. In addition, high blood flow organs exposed to dendrimers, such as kidneys and lungs, may also be affected [129]. However, few studies have investigated the organ-specific toxicity of dendrimers. For the safety of administration, it is necessary to conduct more in-depth toxicity studies on each organ [130]. Specific toxicity studies of each organ will allow us to control the dose and reduce adverse effects in a much more efficient manner.

##### Gastrointestinal Toxicity

When administered orally, dendrimers can enter the gastrointestinal tract and exert toxic effects on the gastrointestinal epithelium [131]. PAMAM dendrimers were shown to penetrate the intestinal epithelium in isolated rat jejunum mounted in chambers, while 1 mM G3.5 and G4 PAMAM dendrimers were unable to induce increased paracellular transport to isolated rat tissue and damaged rat jejunal cells. This difference may have arisen from the mucus and supportive cells underlying the epithelial barrier of the rat jejunal mucosa and their weak sensitivity to permeability when compared with Caco-2 cell culture [132]. Further toxicity studies on PAMAM dendrimers across isolated human intestinal epithelium have also been reported. PAMAM dendrimers at a concentration of 1 mM concentration caused no difference in trans-endothelial cell resistance values in controls. However, at 10 mM, PAMAM G3.5-COOH and G4-NH_2_ dendrimers increased the mannitol permeability and disrupted the surface epithelium in human colonic and jejunal tissues, meaning that high concentrations of dendrimers may induce limited levels of toxicity [119].

##### Hepatorenal Toxicity

Transmission electron microscopy showed that exposure to 100 μg/mL of PAMAM dendrimer G5 for 24 h resulted in the appearance of autophagic vesicles in hepatocytes, hepatocyte necrosis, and vacuolation in mouse liver sections. The administration of two autophagy inhibitors, 3-methyladenine (3-MA) and chloroquine (CQ), resulted in the recovery of liver weight reduction, a reduction in liver tissue injury, and the blockade of serum biochemical parameters in mice when treated with dendrimers, thus suggesting that PAMAM dendrimers may damage liver tissue through autophagy [85]. Jemnitz et al. reported that PAMAM G5 dendrimers induced an increase in calcium that interfered with the calcium homeostasis of hepatocytes in hepatocyte mono- and hepatocyte/Kupffer cell (KC) co-cultures isolated from transgenic rats expressing the GCaMP2 fluorescent Ca^2+^ sensor protein. Furthermore, these authors suggested that this model could be used to predict the hepatotoxic potential of drugs [86].

Nephrotoxicity has a significant effect in many adverse drug responses, due to the central roles of the kidneys in metabolism and blood filtration [92]. A previous study showed that PAMAM G6 dendrimer caused no severe morphological toxicity in the kidneys. However, dendrimers can modulate the signaling of mitogen-activated protein kinases (MAPKs) in vivo through EGFR-independent and oxidative-stress-dependent mechanisms, thus suggesting that a PAMAM delivery system may have important renal toxicological significance [109].

##### Pulmonary Toxicity

The intratracheal administration of PAMAM dendrimer G4-G7 caused an increase in the levels of serum angiotensin II in lung tissue and significantly reduced the survival rate of mice. It was also observed that cationic PAMAM dendrimers could directly bind to angiotensin converting enzyme II (ACE2) protein through surface plasmon resonance. Therefore, Sun et al. hypothesized that the activity and expression of cationic PAMAM dendrimers was reduced by directly binding to ACE2, thus resulting in disorders of the renin–angiotensin system and an increase in ACE2, eventually leading to acute lung injury [116]. Furthermore, the Akt-TSC2-mTOR signaling pathway was found to be a significant mechanism responsible for acute pulmonary toxicity [94].

##### Cardiotoxicity

A previous study of the acute ex vivo or chronic in vivo administration of a G6-PAMAM dendrimer in the heart of healthy non-diabetic rats revealed a deterioration of cardiac-related kinetic parameters and contractility, and an increase in myocardial enzyme levels. These data suggested that the recovery of cardiac function is delayed following cardiac myocardial ischemia-reperfusion (I/R) injury; this probably occurs by inhibition of the EGFR-dependent rescue pathway required for the recovery of cardiac I/R injury [117].

##### Skin Toxicity

Using a rat model, skin irritation tests, following the local administration of PAMAM-NH_2_ G2 and G3 dendrimers (at a concentration of 6 mg/mL), revealed slight erythema and obvious changes in the morphology of epidermal cells. Rats treated with significantly higher concentrations of PAMAM-NH_2_ (30 mg/mL or 300 mg/mL) developed moderate and severe erythema and obvious histopathological changes in the dermis. In addition, when treated with high concentrations of PAMAM-NH_2_ dendrimers, PCNA was highly expressed in all layers of the skin, and the nuclear immune response was enhanced, thus indicating a cell proliferation disorder. Collectively, microscopic evaluations and immunohistochemical analyses showed that cationic PAMAM dendrimers readily led to significant skin toxicity and that low concentrations should be considered for practical external use [118,133].

##### Developmental Toxicity

Sub-lethal concentrations of G4 PAMAM dendrimers with amino functional groups were previously shown to cause the development arrest and mortality of zebrafish embryos. When applied to zebrafish embryos, the toxicity of cationic PAMAM and PPI dendrimers increased over time, eventually reaching 100% mortality (PPI 4.0, only 80% after 96 h), thus indicating that the embryotoxicity of cationic dendrimers had been significantly underestimated; the rate of apoptosis in embryonic cells increased in a concentration-dependent manner [70]. In another study, cationic PAMAM dendrimers exerted an effect on angiogenesis, apoptosis, and certain genes in the early stages of chick embryo development, thus resulting in embryonic toxicity [134].

##### Ocular Toxicity

Dendrimers are highly attractive options for ocular drug delivery systems; thus, the safety evaluation of ocular drug delivery is very important [135]. Previous studies demonstrated that higher concentrations of dendrimers could affect retinal function, without fundus and histopathological changes [90]. Therefore, the ocular administration of dendrimers needs to consider the dosage, to ensure safety.

#### 3.3.5. Genotoxicity

Previous research involving Comet assays revealed that the toxicity of PPI G4 dendrimers was concentration-dependent. When lymphocytes were exposed to high concentrations of PPI G4, the amount of DNA left in the comet’s head decreased, while that in the tail increased, thus indicating that a strong interaction may exist between dendrimers and DNA that can result in strand breaks. Interestingly, when low-dose dendrimers were removed, the DNA–dendrimer complex was removed by the cellular repair system; this led to the disappearance of the toxicity and side effects [97]. A study on the interaction between PAMAM G5 and nuclear material in cancerous and non-cancerous cells with Raman micro-spectroscopy was reported. The reduction in nucleic acid band strength, the reduction in nuclear RNA content, and the reduction in nuclear RNA damage after 24 h, along with DNA modification and cell death, could explain the genotoxicity of PAMAM dendrimers [136].

## 4. Functionalization of Dendrimers 

### 4.1. Reducing Toxicity

The biocompatibility of nanocarriers in a biological environment can be evaluated by investigating both their non-toxic and non-immunogenic characteristics, as well as their degree of stability in the circulation following administration. Thus, dendrimers should be functionalized with biocompatible molecules, in order to enhance their biocompatibility [137]. Various methods can be used to fabricate biocompatible dendrimers for use in a wide range of applications, including cancer therapy. First, the addition of a biocompatible moiety to the dendrimer core; dendrimers with biocompatible cores offer better biodistribution and better circulation, as well as increased surface functionalization capability. Second, the use of branched dendrimers that contain a repeating biocompatible component; these are non-toxic, hydrophilic, and water soluble, rendering them suitable as drug delivery vectors. Third, the surface modification of dendrimers using biocompatible materials; the surface modification of dendrimers with biocompatible materials is an excellent method for reducing toxicity and preventing rapid clearance [138]. Recent studies have shown that the surface engineering of dendrimers can also enhance drug entrapment capacity, drug solubility, biodistribution, pharmacokinetics, and the stability of drugs. The surfaces of dendrimers have been modified by a number of methods, including surface modification with polyethylene glycol, glycosylation, acetylation, amino acids, and peptides; these methods have been used to reduce cytotoxicity and increase drug loading capacity [139].

#### 4.1.1. Poly (Ethylene Glycol)-Modified Dendritic Surfaces

Dendrimers must have a high level of biocompatibility to be used as biomaterials. Dendrimer toxicity is heavily dependent on their terminal groups. For example, PAMAM dendrimers possessing primary amine terminal groups have been shown to be toxic to a number of different cell types [65]. On the other hand, dendrimers with the same backbone, but containing different terminal groups (carboxyl and hydroxyl groups), exhibited a much lower level of cytotoxicity. Based on these findings, it appears that the terminal groups of dendrimers play an important role in controlling their cytotoxicity, mainly because the terminal groups cover the backbone [140]. The functionalization of dendrimers with poly (ethylene glycol) (PEG) chains is an important step in reducing the toxicity of dendrimers towards the endothelium, due to the fact that PEG chains inhibit electrostatic interactions between the cell membrane and the dendrimer surface. The PEGylation of dendrimers refers to the process of conjugating PEG with dendrimers. The functionalization of dendrimers can be achieved in three distinct ways: (i) PEG chains directly conjugated to the surface of a dendrimer; (ii) the attachment of PEG chains to the dendrimer surface with a linker to facilitate drug conjugation with the surface; and (iii) PEGylation of the dendrimer surface using a linker to reduce steric hindrance on the dendrimer surface [141]. Furthermore, the PEGylation of dendrimers may result in improved drug release and biodistribution, as well as the improved penetration of biological membranes, thus enabling the targeted delivery of drugs to specific organs. It has been shown that dendrimer PEGylation improves the solubility and entrapment capabilities of hydrophobic molecules [142]. A previous study demonstrated that PEGylation protects dendrimers from toxicity; the positive charges of PAMAM dendrimers for each of the three groups (G2, G3, and G4) were coated with 200 KDa PEG chains, and the cytotoxic effect of these chains was investigated in Caco-2 cells. The PEGylated PAMAM dendrimers were less toxic than their unmodified counterparts because positive charges had been capped on their surfaces [121]. Based on these findings, PEGylation was suggested to contribute to both reducing the toxicity of drugs and improving the effects of systemic circulation, by preventing their recognition by reticuloendothelial cells [143]. 

G4 PEGylated dendrimers caused 5% less hemolysis in comparison to cationic PAMAM dendrimers. This was due to reduced interaction between cationic groups and cells. In addition, these researchers also observed an increase in the solubility and entrapment efficiency of a hydrophobic substance (5-fluorouracil) [144]. Furthermore, Kojima and colleagues investigated the effect of increasing the molecular weight of PEGs and the generation of dendrimers on dendrimer entrapment and solubility. It was found that hydrophobic chemotherapeutic agents, such as adriamycin and methotrexate, could be successfully encapsulated in dendrimers and that an increase in the PEG molecular weight and generation of dendrimers led to an improvement in drug encapsulation [145]. In another study, Vijayraj et al. reported similar findings for rifampicin delivery with PEGylated PPI dendrimers. In their study, PEGylated PPI dendrimers increased the solubility and entrapment capacity of the drug, while significantly reducing hemolytic toxicity, in comparison to uncoated PPI dendrimers [146]. Therefore, PEGylated dendrimers appear to be capable of increasing the solubility of hydrophobic compounds and encapsulation efficiency but also minimize the unwanted characteristics of PAMAM dendrimers.

Dendrimers with amine-terminated chains can be attached to PEG chains by reaction with methoxy-PEG-4-nitrophenyl carbonate. Different types of dendrimers have been shown to have the ability to encapsulate small molecules. A number of studies have shown that the PEGylation of PAMAM dendrimers results in the formation of stealth-type dendrimers with good levels of biocompatibility [147]. This suggests that as compared to native PAMAM dendrimers, PEG PAMAM dendrimers can enhance the viability of endothelial cells by approximately 40% after 24 h of incubation. Jacobson et al. compared G3 amine-terminated PAMAM dendrimers conjugated with PEG groups of different molecular weights (Mn 14,550, 750, and 2000) with N-acetylated types of the same dendrimers. The results of this study indicated that the PEGylated dendrimer conjugates (PEG Mn 1450, 750) were two- to nine-fold less toxic than those containing N-acetylation but no PEGylation. In addition, PEGylation increased the biodistribution and pharmacokinetics behavior, as well as drug loading capacity [148]. Nevertheless, the conjugation of PEG chains on the surfaces of dendrimers can be influenced by the length of the PEG chains, and the number of arms in the chains; this affects the amounts of drug that can be encapsulated in the cavities of the dendrimers. Kojima and colleagues encapsulated anticancer drugs in PEGylated dendrimers and demonstrated that longer PEG arms resulted in a greater loading capacity, as well as an increased stability of the entrapped molecules [145].

#### 4.1.2. Acetylated Dendrimers

The acetylation of dendrimers is another effective method of reducing their toxicity; this is because the acetyl group is conjugated with the terminal group, thus neutralizing the positive charges on the surface of the dendrimers. Acetylated dendrimers exhibit higher water solubility; this is important for drug development and bioapplications [65]. Zhuo et al. synthesized and characterized dendritic polymers containing 1, 4, 7, and 10 tetra-aza-cyclododecane; subsequently, the functional groups were acetylated with acetic acid. These findings supported the conclusion that acetylated dendrimers promote better drug solubility and ensure that drug delivery occurs in a controlled manner [149]. Similarly, Waite et al. investigated the delivery of siRNA to cells through acetylated dendrimers using PAMAM G5 dendrimers. These researchers synthesized PAMAM dendrimers that were acetylated with acetic anhydride and then complexed with siRNA; the cytotoxic effects of these dendrimers were then tested on U87 glioma cells. The authors found that acetylation of the PAMAM dendrimers led to a significant reduction in cytotoxicity and a significant increase in the rate of dissociation and siRNA release [150]. In another study, Kolhatkar et al. reported that the acetylation of dendrimers alters their cytotoxicity, cellular uptake, and permeability, without affecting their intracellular permeability [151]. Wang et al. prepared acetylated PPI dendrimers with different concentrations of acetyl groups (14.2–94.3%), and subsequently encapsulated sodium deoxycholate, methotrexate sodium, and doxorubicin within these dendrimers. Interestingly, the acetylated PPI dendrimer significantly reduced cell viability in A549 and MCF-7 cells. A higher acetylation ratio (>80%) was correlated with a greater drug loading capacity, decreased toxicity, and an improved pharmacokinetics profile. The cytotoxicity of doxorubicin and methotrexate sodium decreased significantly when they were complexed with acetylated PPI dendrimers with high degrees of acetylation [152]. In another study, Majoros et al. used a variety of techniques to determine the precise proportion (%) of acetylation, including potentiometric titration, gas chromatography, and nuclear magnetic resonance (NMR). Specifically, these researchers determined the exact stoichiometric ratios required for acetylation, as well as the role that acetylation played in the synthesis of PAMAM G 5.0 dendrimers [153]. Similarly, Kukowska-Latallo et al. did not observe any morphological changes when examining the liver, kidney, lungs, or heart following acetylation. 

This implies that acetylation can be effectively utilized to reduce the cytotoxicity of a drug and to increase its efficacy with regards to dissolution, permeability, and uptake by the body [154]. The acetylation process has several advantages over PEGylation, including (i) ease of use and high efficiency, (ii) low levels of acetylation, when choosing a suitable ratio between acetic anhydride and dendrimer, and (iii) low steric hindrance during acetylation, when compared with the PEG chain. Moreover, acetylated dendrimers cross cell membranes and maintain permeability, whereas PEGylated dendrimers exhibit a low cellular uptake [141]. 

Stasko et al. studied the cytotoxicity of unlabeled and fluorescently-labeled acylated G5 PPI dendrimers in cultured human umbilical vein endothelial cells (HUVECs) and their effects on membrane disruption. According to confocal fluorescence microscopy, the dendrimers containing acetyl groups exhibited significantly lower levels of cytotoxicity when compared to plain PPI counterparts. Consequently, the acylated G5 PPI dendrimers had no adverse effects on HUVEC cells under experimental conditions [155].

Zhuo et al. successfully developed acylating PAMAM dendrimers to deliver drugs in a controlled manner. These researchers synthesized dendrimers consisting of a core of 1, 4, 7, and 10 tetraazacyclododecane followed by Michael addition reaction of methyl acrylicate and subsequent amidation. Acetic anhydride was used to acylate the dendrimers. Dendrimer-5 FU conjugates were subsequently synthesized from the acylated dendrimers, by treating them with 1-bromoacetyl-5-fluorouracil. Due to the acylation reaction, the PAMAM dendrimers became highly soluble and, upon hydrolysis, released 5-fluorouracil, which can then exert therapeutic effects. Specifically, 5-flurouracil was released in a controlled manner, thus reducing its cytotoxicity, while also proving useful for delivering chemotherapeutic drugs [149]. 

In a similar study, Kukowsaka et al. developed acylated PAMAM dendrimers as delivery vehicles for methotrexate to human KB tumors in mice that expressed the folic acid receptor. In this study, folic acid conjugated and acetylated PAMAM dendrimers were used to conjugate methotrexate or tritium with fluorescein or 6-carboxytetramethylrhodamine, which was then injected into animals to test the effect of the drug. Based on a histopathological investigation conducted over 90 days following administration, no toxicity was observed and no abnormalities were noted in the liver, kidneys, lungs, spleen, or heart [154]. Acetylated and glycidylated bifunctional dendritic system was synthesized by partial acetylation of the dendrimer, labeling with FITC, linking of folic acid via an amide bond, glycolysis of the remaining amino groups, and ester linking of the chemical drug to the dendrimer. This revealed that all dendrimer conjugates had a very narrow molecular weight distribution and a polydispersity below 1. As a result of this approach, succinic linkages were established between the drug and glycidylated dendrimers via double ester linkages, which are susceptible to enzymatic and acidic degradation and are ideal for studying cell absorption [156]. Dendrimers containing acylated groups may be a promising alternative to the traditional methods and current systems used for drug delivery.

#### 4.1.3. Anionic or Half-Generation Dendrimers

Generally, full-generation PPI and PAMAM dendrimers have peripheral polycationic functional groups, while half-generation dendrimers are characterized by cyanide or carboxylic acid reactive groups on their surfaces. According to previous studies, the toxicity of dendrimers is only caused by polycationic charges on the surface of the material. Therefore, research is focused on half-generation dendrimers, to improve biocompatibility. In contrast, dendrimers with negative charges or groups on their surfaces were found to exert negligible toxicity or hemolytic effects [157]. Malik et al. carried out in vitro cytotoxicity studies in cell lines, as well as in vivo biodistribution studies of full-generation and half-generation PAMAM dendrimers in mice, with the intent of comparing their effects. The results suggested that half-generation PAMAM dendrimers were relatively non-toxic up to a concentration of 2 mg/mL, whereas full-generation PAMAM dendrimers induced hemolysis upon exposure at a concentration of 1 mg/mL. On the other hand, cationic dendrimers appear to clear more quickly from the body (2% of the dose was recovered from the blood after one hour). Half-generation PAMAM dendrimers appeared to clear at a much slower rate (between 20% and 40% recovery from the blood at one hour) compared to cationic PAMAM dendrimers [73]. Similarly, Bhadra et al. reported that half-generation PAMAM dendrimers exhibited significantly less hemotoxicity. During in vitro experiments, cationic PAMAM dendrimers exhibited 15.3–17.3% hematotoxicity, whereas half-generation PAMAM dendrimers did not appear to have any adverse effects on blood cells [144]. In vitro cytotoxicity studies were performed in CaCo-2 cells by Sohant et al., using full- and half-generation PAMAM dendrimers. In contrast to full-generation cationic PAMAM dendrimers, the half-generation PAMAM dendrimers did not induce cytotoxicity. This suggests that PAMAM and PPI dendrimers containing carboxylic acid or cyanide terminal groups are not cytotoxic and can be used safely as therapeutic vehicles [143].

#### 4.1.4. Glucoheptoamide Dendrimers

According to one study, a third-generation poly(amidoamine) dendrimer was amidated stepwise using d-glucoheptono-1,4-lactone. Glucoheptoamidation (up to 1/3 of the amine groups of neat dendrimers available) was found to increase conjugate size and ζ potential. Conjugates of native or low substituted dendrimers accumulated efficiently in fibroblast cells at a concentration of 1 µm, which was nontoxic. In addition, increased substitution of the dendrimer resulted in a consistent decrease in the cell’s size, zeta potential, and toxicity. A modified dendrimer, half filled with glucoheptoamide substitutes, was employed as a carrier for covalently bound cytisine molecules, a rigid alkaloid, and a hydrophobic alkaloid. The conjugate containing two cytisines and sixteen glucoheptoamide substituents accumulated rapidly and was not toxic up to a concentration of 200 µm. Half-glucoheptoamidated PAMAM dendrimer is being considered as a potential anticancer drug carrier [158].

#### 4.1.5. Dendrimers Conjugated with Amino Acids or Peptide Groups

Amino acids and peptides are molecules produced by the human body that are biocompatible and safe to use in human tissues. Dendrimers tagged with phenylalanine and glycine have shown a marked reduction in toxicity. Glycine and phenylalanine are conjugated with dendrimers by amino acid conjugation. The use of these amino acids may help to reduce the potential toxicity of dendrimers, such as cytotoxicity, hemotoxicity, and immunogenicity. In their study, Kono et al. synthesized and modified PAMAM G 5.0 dendrimers with arginine, phenylalanine, and leucine by surface conjugation and compared the efficacy of their gene transfection efficiency. All of these amino acid-conjugated G 5.0-PAMAM dendrimers formed a major histocompatibility complex (MHC), which had a better immunostimulatory capability [134]. These studies suggest that amino acid tagged PAMAM dendrimers can serve as potential candidates for DNA vaccines, due to their high transfection and targeting abilities [60].

Dendrimers that are conjugated with amino acids are known to be less toxic. Agashe et al. compared the cytotoxicity, hematotoxicity, and immunogenicity of glycine/phenylalanine conjugated G5 PPI dendrimers to cationic dendrimers. Their results showed that dendrimers containing amino acids were not cytotoxic or hepatotoxic (depending on the generation and dose used), and also showed that dendrimers that are modified in this manner are safe to use in biological systems [53]. Kono et al. revealed that amino acid terminated PAMAM dendrimers were also capable of delivering genes. As a result of the hydrophobic properties of these gene carriers, they interacted successfully with cells, thus resulting in the efficient transfection of genes into cells. It has been demonstrated that dendrimers have a higher transfection rate when it comes to delivering genes to cells. Thus, these authors developed G4-specific PAMAM dendrimers containing leucine or phenylalanine groups to facilitate gene transfer. PAMAM dendrimers that had been modified with phenylalanine or leucine had an enhanced transfection rate with reduced cytotoxicity [159]. Yang and Kao produced arginine-glycine-aspartate peptides and coupled them to PAMAM cationic G 4.0 and anionic G 3.5 dendrimers. These authors then investigated the cellular internalization of these conjugated dendrimers in adherent fibroblasts and determined that anionic G3.5 PAMAM peptide complexes did not negatively affect fibroblast viability at higher concentrations, while cationic G4.0 complexes did so at lower concentrations [160]. One previous study described the coupling of the follicle stimulating hormone (FSH) peptide domain with fluorescein labelled G5 PAMAM dendrimers. In addition, these complexes showed a high degree of receptor specificity for human ovarian cancer cells (OVCAR-3), which are known to express the FSH receptor. Furthermore, these complexes resulted in significant uptake of the drug, as well as the downregulation of a protein that inhibits apoptosis [161]. Therefore, when modified with amino acid groups, PAMAM and PPI dendrimers have significant potential as vehicles for the delivery of a wide range of drugs and biological agents, to treat a variety of diseases with minimal or no cytotoxicity. 

In addition to amino acids, some peptides have also been conjugated to dendrimers. In recent times, a peptide–dendrimer was developed by Kojima et al. for the targeting of leukemia cells in the treatment of acute myelogenous leukemia. Cathepsin B was linked to the scaffold, in addition to using CPP44 peptide as a tumor-targeting ligand. The results indicated that the scaffold led to better tumor penetration and improved antitumor effects [162]. Researchers reported the development of a dual-targeting nanocarrier system for delivery to bone, designed by covalently attaching a C11 peptide and CH6 aptamer on the G4.0 PAMAM dendrimer (CH6-PAMAM-C11). According to their in vivo biodistribution analysis, CH6-PAMAM-C11 accumulated rapidly in bone after 4 and 12 h, and delivered drugs to sites of osteoblast activity. CH6-PAMAM-C11 components were easily excreted through the kidneys [163]. The conjugation of dendrimers with peptides has yet not been extensively investigated, but may offer a window of hope for improving the safety profiles of dendrimers.

#### 4.1.6. Dendrimers Conjugated with Drugs/Genes/DNA

Dendrimer conjugation, along with dendriplex formation, is also known to mask the cationic charge. The tagging of bendamustine on PAMAM G4.0 dendrimers led to a reduction of hemotoxicity by several fold. Kolhatkar et al. reported improved levels of cellular uptake and reduced toxicity in the presence of drug-conjugated PAMAM dendrimers. These authors conjugated dendrimers to 7-ethyl-10-hydroxy-camptothecin (SN-38), a very potent topoisomerase I poison, to the active metabolite of irinotec anhydrochloride (CPT-11). SN-38 is a compound 1000 times more potent than CPT-11, whereas irinotecan is less water soluble, has insufficient oral bioavailability, and causes gastrointestinal side effects. For this reason, the authors conjugated SN-38 with G4PAMAM dendrimers to improve its oral bioavailability. Then, they studied the stability, uptake by cells, permeability, and toxicity of the compound using CaCo-2 cells. In addition to enhancing cellular uptake, the conjugation of SN-38 with G4 PAMAM dendrimers led to reduced toxicity and water solubility. The study showed that the SN-38 conjugated PAMAM dendrimers were stable (pH 7.4) and retained the drug (>80%) within two hours of conjugation [164]. G4 PAMAM dendrimers demonstrated minimal cytotoxicity in terms of in vitro cytotoxicity experiments in CaCo-2 cells. Following two hours of incubation with drug-conjugated PAMAM dendrimers at concentrations of 0.1 µM, 1 µM, and 100 µM, more than 90% of CaCo-2 cells remained viable. Therefore, this drug-conjugated G4 PAMAM dendrimer improved cellular uptake and stability with a non-toxic profile. The authors showed that these dendrimers could be used as carriers to deliver the SN-38 anticancer agent to cancer cells. Similarly, in another study, Lee et al. conjugated doxorubicin with a G3 hybrid PEO-dendrimer. This conjugation was evaluated for toxicity, both in vitro and in vivo, and compared to free doxorubicin treatment. The authors found that the conjugation of this dendrimer with doxorubicin proved to be 10-fold less toxic to colon cancer cells after 72 h, as compared with the treatment with free doxorubicin. Based on in vivo animal tests performed on a BALB/c mice model with subcutaneous C-26 tumors, it was found that drug-conjugated dendrimers exhibited nine-fold greater cellular uptake of the drug compared to the free drug. The authors also observed that 100% of the animals survived after being administered with 2 mg/kg of drug-conjugated dendrimers intravenously [44]. In another study, Li et al. synthesized two hydrophilic hydrophobic poly (L-lactide) blocks D2 and a biodegradable hydrophobic poly(L-lactide) block LLA15, which served as biodegradable carriers for gene transfer. These biodegradable carriers possessed much higher affinities for DNA than other hydrophilic carriers, as well as considerably less cytotoxicity [165]. In vitro experiments conducted on SMMC-7721 cells also revealed a 10-fold higher rate of gene transfection. Thus, dendrimers conjugated with drugs or genes may prove useful for treating a variety of diseases.

### 4.2. Improving Targetability

#### 4.2.1. Antibody-Functionalized Dendrimers

Novel therapeutic approaches, such as antibody-conjugated delivery systems, may be used to facilitate the delivery of cytotoxic and other therapeutic agents to targeted tissues. These antibodies serve as a means for the immune system to target tumor cells that produce specific antigens. These antibody-conjugated dendrimers affect the vessels and tissues in the body that nourish and support the development of tumor cells. In addition, these compounds prevent tumor growth, by blocking the growth factors that are essential for tumor growth. This phenomenon is known as an anti-angiogenic effect [166]. As a result of their low immunogenicity and low water solubility, PAMAM dendrimers have been demonstrated to be excellent carriers for antibodies. Shukla et al. demonstrated the targeted delivery of anti-human epidermal growth factor receptor-2 monoclonal antibodies (HER2 mAb) conjugated to G5 PAMAM dendrimers. HER2 monoclonal antibodies were conjugated to G5 PAMAM dendrimers, which were then labeled with Alex Fluor. Next, the conjugates were used to treat cancer cells that overexpressed human growth factor receptor-2, such as breast and ovarian cancer cells. These antibody-conjugated dendrimers were observed to exhibit enhanced antibody binding, as well as enhanced internalization of the conjugates into MCA-207 cells. Studies in animals showed that the conjugates were capable of delivering chemotherapeutics to tumor cells that overexpressed HER2 [167]. Wangler et al. tested the effect of labelled antibodies on immunoreactivity in terms of the size and degree of conjugation. These authors used chelating agents to combine an anti-EGFR antibody, hMAb425, with a new generation of PAMAM dendrimers. The results showed that the immune reactivity of the cells was significantly influenced by the number of derivatization sites, whereas the size and type of dendrimer did not have a significant impact on the effectiveness of targeting [168]. Patri et al. also reported the synthesis and in vitro evaluation of G5 PAMAM dendrimers conjugated with monoclonal antibodies against PSMA expressed on prostate epithelial cells. Flow cytometric analysis of the conjugated dendrimers and antibodies revealed that the conjugated dendrimers and antibodies had a specificity for PSMA-positive LNCaP.FGC cells. In this study, the conjugates failed to bind to PC-3 cells, which cannot express PSMA. Confocal microscopy indicated that the conjugates of dendrimers are more effective at binding to antibodies and promoting cellular internalization than the free dendrimers [169]. Furthermore, encapsulating gold nanoparticles in PAMAM dendrimers and tailoring them further with Herceptin Ab demonstrates that this nano-assembly had a selective binding affinity for HER-2 overexpressing cells, whereas the unmodified nano-assembly had no affinity for HER-2 cells [58]. Cetuximab, an anti-EGFR monoclonal antibody, was also covalently tethered to PAMAM G5.0 dendrimers and further loaded with methotrexate, an antineoplastic agent. In vitro testing demonstrated that the conjugates exhibited significant cytotoxicity to F98 cells and had a high binding affinity [170]. Previously, Xie et al. conjugated a Slex Ab to PAMAM dendrimers, which were then characterized. The authors concluded that in HT29 cells, the modified dendrimers were taken up more readily than when unmodified, although this uptake was dependent on the concentration used [57]. A PAMAM-trastuzumab conjugate loaded with doxorubicin was recently developed by Marcinkowska et al., to further augment the efficacy of this therapy. According to the authors, this novel conjugate was capable of selectively binding to cells overexpressing HER-2. However, this conjugate exhibited a considerably higher cytotoxicity than the free drug against cancer cells [171]. Thomas et al. conjugated 60 anti-bCA and J591 antibodies to PAMAM G 5.0 dendrimers and determined the efficiency at which these antibodies targeted both CD 14+ (HL-60 human myeloblastic leukemia cells) and prostate cancer cells (LNCaP). The 60bca antibodies and J591 antibodies were labelled with fluorescein isothiocyanate (G5-FI-60B, G5-FI-PA), to allow detection of the dendrimer. Flow cytometry, confocal microscopy, and two-photon-based optical fiber fluorescence (TPOFF) detection systems were used to evaluate the dendrimer-antibody conjugations [172]. The authors demonstrated that antibody-conjugated nanocarriers significantly enhanced the uptake and targeting efficiency of antigen-expressing cells. Consequently, antibody-conjugated dendrimers have the potential to exhibit improved cellular uptake and cellular internalization of therapeutic agents.

#### 4.2.2. Folate-Conjugated Dendrimers

Folic acid receptors are overexpressed in many types of tumor cells, such as those found in the kidney, lung, ovary, endometrium, colorectal, mammary, breast, testis, erythrocytes, neuroendocrine carcinomas, and brain metastases. Thus, receptor-mediated endocytosis facilitates the penetration and uptake of folic acid-conjugated nanoparticles into tumor cells and increases targetability [173]. In this sense, folic acid-conjugated nanocarriers might be effective in cancer-targeting therapies. Moreover, dendrimers that have been modified with folic acid on their surfaces may function effectively as a gene delivery agent, imaging agent, and diagnostic agent [174]. Choi et al. conjugated folate to partially acetylated PAMAM dendrimers and produced a complex that covalently adhered to DNA and generated a DNA-linked dendrimer cluster. This conjugated DNA-linked dendrimer cluster was found to internalize and specifically target KB cells in an in vitro study overexpressing the folate receptor [175]. In another study, Singh et al. compared the efficacy and safety of folic acid- and folic acid-PEG-conjugated PAMAM dendrimers in tumor-bearing mice. The authors found that the folate-PEG dendrimer-conjugate was non-toxic in terms of hemolysis and effective in targeting cancer [176]. In another study, Kono et al. formulated polyether dendrimers for targeted drug delivery [177]. These dendrimers were synthesized by converting esters to hydrazides through reaction with hydrazine; folate groups were then conjugated to dendrimer hydrazides. Dendrimers conjugated with folate have a higher solubility in phosphate buffers at physiological pH values, as well as more hydrophobic functional components; these characteristics enable the folate-overexpressed cells to encapsulate hydrophobic drugs. Folate residues on the surface of dendritic cells have been shown to improve the targeting of anti-tumor drugs to tumor cells that express folate receptors. In addition, Naarmani et al. modified PAMAM G 4.0 dendrimers with PEG, so that they would have a reduced cytotoxic effect. Dendrimers that were partially PEGylated were modified with folic acid, in order to develop nanocomplexes to target C2C12 myoblasts [178]. Quintana et al. developed a G5 PAMAM dendrimer by conjugating fluorescein isothiocyanate and subsequently reacting this with folate using 1-ethyl-3-(3-dimethylaminopropyl) carbodiimide [179]. In another study, the anti-cancer drug methotrexate was shown to be highly effective in targeting cancer cells with reduced toxicity when loaded into PAMAM G5.0 dendrimers [177]. Therapies targeted to lymphatic tumors require both hydrophilicity and hydrophobicity. In addition, these PAMAM dendrimers have a folate-conjugated form, which allows hydrophilic drugs to penetrate the cellular barriers and reach cancer cells in tumor xenografts. Thus, these studies showed that folate conjugated dendrimers have excellent biocompatibility and targeted drug delivery properties.

#### 4.2.3. Carbohydrate-Engineered Dendrimers

Glycodendrimers are dendritic structures that contain carbohydrates. These glycodendrimers can be classified based on the distribution of carbohydrates in the dendritic structure. For example, those modified with carbohydrates at the center, base, and surface. Cationic dendrimers coated with carbohydrates exhibit reduced hemolytic toxicity, reduced immunogenicity, and enhanced targeted drug delivery in comparison to uncoated dendrimers [180]. Bhadra et al. successfully delivered primaquine phosphate to liver cells with dendrimers that were conjugated with galactose. Other researchers synthesized galactose-coated PPI dendrimers and compared their efficacy, hematological toxicity, and cytotoxicity with dendrimers without galactose coatings [181]. Galactose is one of several ligands that are capable of binding to the asialoglycoprotein receptors on liver cells. As a result, drugs can be delivered directly to liver cells by the application of galactose-conjugated dendrimers. Bhadra et al. reported that PPI dendrimers coated with galactose increased drug entrapment by 5–15-fold or more, when compared to uncoated PPI dendrimers. Dendrimer coatings produced a sustained release of the drug, as opposed to uncoated dendrimers. The galactose-coated dendrimers also demonstrated reduced hematotoxicity. G4 and G5 conjugated galactose dendrimers accounted for 10% and 7.1% of toxicity, respectively; while uncoated G4 and G5 PPI dendrimers accounted for 35.4% and 49.2% of toxicity, respectively [181]. In another study, galactose-coated dendrimers were synthesized by Agrawal et al. to deliver chloroquine phosphate, without causing harmful side effects when administered intravenously. Dendrimers that were coated with galactose were compared with those not coated, with regards to their effect on hematological parameters. According to the results, when galactose coated G4 and G5 dendrimers were used at a concentration of 5 mg/mL, they lowered RBC hemolysis to 6% and 7.2%, respectively, compared to that of uncoated G4 and G5 dendrimers. In addition, galactose-coated dendrimers demonstrated fivefold less phagocytosis in macrophages, when compared with uncoated dendrimers [180]. These results indicated that the coating of dendrimers with galactose could reduce their hemolytic toxicity, thus making them more suitable as delivery systems for therapeutic agents.

#### 4.2.4. Tuftsin-Conjugated Dendrimers

Tuftsin is a tetrapeptide (Thr-Lys-Pro-Arg) that is involved in a variety of immune-related biological functions and exhibits a wide range of immunoactivities, including migration, bactericidal, phagocytosis, and tumoricidal properties. Tuftsin is produced by the enzymatic degradation of the heavy chain of immunoglobulin G within the fragment crystallization domain. Due to its structural similarity, Tuftsin specifically binds to macrophages, polymorphonuclear leukocytes, and monocytes. Consequently, the conjugation of this molecule is of great importance in the targeted treatment of HIV, as well as improving the efficacy of chemotherapy in tumors with reduced systemic toxicity [182]. Najjar et al. demonstrated that a fraction of gamma-globulin stimulated phagocytosis by specifically binding to neutrophilic leukocytes and monocytes. The mechanism underlying this activity was demonstrated by the use of tetrapeptide (residues 289 to 292 of the heavy chain of leukokinin), which is also known as tuftsin (threonyl-lysyl-prolyl arginine) [183]. Only the free tetrapeptide form of this compound exerts an activity. The leukokinin protein chains are cleaved into free forms by two enzymes: Tuftsin-endocarboxypeptidase (found in splenic tissue) and leukokinase (found on the surface of phagocytic cells). Considering its specific binding to leukocytes, macrophages, and monocytes, and the fact that it has reduced toxicity, this peptide makes an excellent carrier for targeted drug delivery. For example, Fridkin et al. conjugated tuftsin with azidothymidine for the targeted drug delivery to HIV-infected macrophages. Analysis showed that reverse transcriptase and HIV antigen expression were inhibited. In addition to stimulating IL-1 release from macrophages and enhancing their immunogenic function, these conjugates were non-toxic to T-cells, thus suggesting that they may be used for the treatment of AIDS [184]. In a similar manner, another group developed efavirenz-loaded and tuftsin-conjugated G5 PPI dendrimers to deliver efavirenz to HIV-infected macrophages. The PPI dendrimer conjugates exhibited a 49.31% drug entrapment efficiency, while the PPI dendrimers exhibited only 37.43% [185]. Furthermore, the tuftsin-conjugated PPI dendrimers exhibited significantly less cytotoxicity in comparison with PPI dendrimers. In addition, the former exhibited a 19-fold higher cellular uptake of efavirenz when compared to the PPI-dendrimers. Dutta et al. analyzed the toxicological profile of G 5.0 PPI dendrimers by means of the surface functionalization of its structure. The RBC count and hemoglobin levels decreased at a low dose of PPI, thus suggesting hemolytic toxicity; however, the tufsin-conjugated PPI did not appear to cause degeneration, immunogenicity, or hemolysis [185]. Consequently, these findings showed that tuftsin-conjugated surface-engineered PPI dendrimers improved biocompatibility and reduced hemolysis. In a follow-up study, tuftsin-conjugated PPI G 5.0 dendrimers (TuPPI) were prepared using efavirenz, to test their efficacy against HIV-infected macrophages in vitro. The uptake of Efavinz with TuPPI was dramatically increased when compared to the free drug. TuPPI has been investigated in macrophage cell lines, to determine its cytotoxicity. The analysis showed that because TuPPI contains polycationic and cytotoxic amino groups that are shielded by bulky peptides, the cytotoxicity of modified PPI dendrimers is significantly reduced when compared with unmodified forms. Thus, dendrimers conjugated to tuftsin possess therapeutic potential, due to their ability to enhance cellular uptake, minimize cytotoxicity, and act as efficient carriers of genes and drugs inside the body.

#### 4.2.5. Alpha-Mangostin (αM)-Conjugated Dendrimers

Biotin transport-targeting polysaccharide-modified PAMAM dendrimers were synthesized by bonding αM with the surface amine groups of dendrimers through amide bonds in the ratio of 5 (G3^2B12gh5^) or 17 per dendrimer molecule (G3^2B10gh17M^). The remaining 12 or 10 amine groups were modified using D-glucoheptono-1,4-lactone (gh) as a blocking agent and two biotin residues as targeting agents. Dendrimer vehicle G3^2B12gh^ had no antiproliferative effect on glioma U-118 MG and squamous cell carcinoma SCC-15 cancer cells. A significant increase in cytotoxicity was observed when αM was attached to the vehicle, improving the observed cytotoxic effect by 4- and 25-fold for G3^2B12gh5M^ and G3^2B10gh17M^, respectively. In both cases, the bioconjugates were efficiently absorbed by the cells. The proposed α-mangostin delivery system provided more efficient delivery of the drug in the dendrimer-bound state compared to the free state in both cancer cells and model organism, which indicated that this treatment would be effective against both cancer and nematodes [186]. In addition, the same group reported that G3 PAMAM dendrimers modified with 16 gh residues were nontoxic up to 100 µm [158]. These results confirmed the effectiveness of biotinylated and partially glucoheptoamidated PAMAM G3 dendrimer as a smart drug delivery platform. 

## 5. Dendrimers for the Diagnosis of Disease

### 5.1. Contrast Agents

The basic principles of MRI imaging relate to the arrangement and relaxation of magnetic nuclei in an external magnetic field. Contrast agents act by shortening the T1 or T2 relaxation time, thus resulting in a light-dark contrast to generate contrast images [187]. Gd-based chelate is a commonly used T1 contrast agent that has a low molecular weight and stays in the blood only for a short time; therefore, this agent cannot generate clear contrast images [188]. In addition, these low molecular weight contrast agents must be administered in high doses to form clear images. However, high doses of chelates will cause metal toxicity. Therefore, dendrimers were developed as new contrast agents. In 1994, Wiener et al. covalently connected the chelator 2-(4-isothiocyanatobenzyl)-6-methyl-diethylenetriaminepentaacetic acid with the free amine on PAMAM via a thiourea bond to form PAMAM-TU-DTPA [189]. Then, GdCl_3_ (III)(pH = 5) was added to PAMAM-TU-DTPA (pH = 5.7) to prepare a new contrast agent, PAMAM (G6)-TU-DTPA-Gd (III). The molecular weight of the new contrast agent was approximately 139,000 g/mole and the ionic relaxation was 34 ± 4 (mM·s)^−1^, at 25 MHz, pH 7.4, and 20 °C; the ionic relaxation of Gd-DTPA alone was only 5.4 ± 0.3 (mM·s)^−1^ under the same conditions. In addition, compared with the half-life (24 ± 4) _min_ of Gd-DTPA, the new contrast agent significantly prolonged the half-life (200 ± 100) _min_. In addition, these authors also found that one G6 PAMAM-TU-DTPA molecule saturated with Gd(III) was equivalent to 1074 Gd(III)-DTPA molecules. In vivo experiments showed that the images generated by an injection of the novel contrast agent at a dose of 0.05 mmole/kg showed a greater number and higher definition of blood vessels than a dose of 0.1 mmole/kg dose of Gd(III)-DTPA [189]. The application of this new contrast agent significantly enhanced the contrast and represents a key step in the future of visual medicine. Contrast agents based on dendrimers can increase the residence time of low molecular weight chelates (GD) in the blood, to obtain much clearer images. In addition, the multivalent surface of dendrimers can significantly improve the relaxation of contrast agents and reduce the dosage of Gd, thus reducing the risk of metal poisoning in the human body [39]. Furthermore, macromolecular contrast agents can be used to passively target contrast agents to tumors [190]. At present, a variety of contrast agents have been developed that are based on dendrimers [191,192,193,194]. Chelate contrast agents based on dendrimers are a new type of contrast agent with significant potential. It is believed that more novel and comprehensive contrast agents based on dendrimers will be developed and available in the near future.

### 5.2. Molecular Probes

Based on the special structural properties of dendrimers (such as the large number of functional terminal active groups), sensors can be modified into the rich surface groups of dendrimers, to form a class of special molecular probes [12]. In 2006, Talanov et al. took PAMAM(G6) ethylenediamine (EDA) as the core and connected bifunctional chelating agents (1B4M-DTPA), NIR dyes (Cy5.5), and GD(III.) to G6, in turn, via covalent bonds, and successfully prepared a novel bimodal nanoprobe that was referred to as G6-(Cy5.5)1.25-(1B4M-GD)145 (suitable for both MRI and fluorescence imaging) [195]. Subsequently, these researchers used the prepared contrast agent as an evaluation object for the sentinel lymph nodes of mice and injected the agent into the breast fat of normal mice to investigate its imaging ability. The authors found that this novel G6 dendrimer-based nanoprobe achieved effective visualization of the sentinel lymph nodes and lymphatic drainage. G6 dendrimer-based contrast agents generated sharper images when compared to high doses of GD(III) alone [195]. A molecular probe based on the fifth generation PAMAM (G5) was developed in 2019 for the real-time monitoring of T cells [196]. Gold nanoparticles were added internally for CT imaging, while fluO-4, a calcium signal sensor molecule covalently modified by ester bond, was added externally to facilitate fluorescence imaging. Thus far, this method has shown good results in the treatment of some solid tumors. Over recent years, miRNAs have been particularly notable as new biomarkers for the diagnosis and treatment of cancer; however, this relies on whether miRNAs can be accurately detected [197]. In a recent study, Liu et al. successfully prepared a novel probe referred to as RP8-MAP4-DNA (D-based NA-peptide tree macromolecules) that can amplify a target miRNA signal by more than eight-fold [198]. The combination of this probe and LC-MS/MS provides us with a visual strategy to fight tumors.

### 5.3. Immunosensors

Traditional immunoassay methods such as ELISA and FLISA are complex, time-consuming, and uneconomical. An immune sensor is a form of biosensor composed of a recognition component (antibody) and a transduction component (output signal) [199]. Dendrimers, due to their unique structure and high concentration of peripheral active groups, can improve the sensitivity of a sensor by controlling the direction of the antibody on the sensor surface. When an antigen binds to an antibody, it produces a signal that is picked up and measured by a sensor; therefore, the target analyte, the antigen, is detected. Therefore, it is evident that dendritic-based immunosensors have significant potential for development and application. Recently, Akter et al. successfully prepared an immunosensor based on 3,3′,5,5′-tetramethylbenzidine-carboxylic acid dendrimer, a label-free immunosensor used to detect cardiac troponin I (TnI) [200], which is the main biomarker used for diagnosis of acute myocardial infarction [201]. These results show that this new immunosensor has a low detection limit, signal selection, and strong stability. A range of different immunosensors are being developed for different detection antigens, such as hepatitis B virus (HBs), prostate-specific antigen (PSA), 5-hydroxymethylcytosine (5hmc) in tumor tissue genes, and α-1-fetoprotein [202,203,204,205,206,207,208]. The further development of dendrimer-based immunodetectors that are novel, more precise, highly selective, and with low levels of toxicity is currently ongoing. 

## 6. Prospects

The poor efficacy of conventional drug carriers and biologicals has prompted the development of novel approaches, to enhance the delivery of drugs and other substances to inaccessible sites in the body, while minimizing their unwanted distribution and toxicity to healthy tissues. Novel approaches that involve nanoparticles loaded with drugs offer unprecedented possibilities, both at a preclinical and clinical level. The efficacy of nanodrug delivery systems in vivo is still being debated, especially with regards to their targeting, distribution, biocompatibility, and efficacy; these factors are crucial for effective diagnosis and treatment. Dendrimer-based therapeutics have emerged as a promising alternative to the conventional nanocarriers that are currently available for treating various diseases. There are several properties that distinguish dendrimers from other nanocarriers, including highly branched structures, clearly defined molecular weights, globular forms, and their small size. Based on their structural properties, different types of dendrimers can provide novel platforms to deliver various therapeutic agents. For example, the fact that we can control their branching length, size, and shape, as well as the flexibility in design, renders dendrimers perfect candidates for applications in nanomedicine, such as drug delivery. Due to their unique structural organization, drugs may be incorporated into dendrimers or conjugated with dendrimers via molecular interactions in different locations. Dendrimers improve the permeability, bioavailability, biocompatibility, and solubility of drugs. Dendrimer nanostructures have attracted considerable attention for their use in the delivery of drugs and in the diagnosis of disease. There has been a substantial increase in patents and scientific publications relating to dendrimer-based nanomedicines over the last few years. Many of these nanomedicines have been commercialized or are currently being tested in clinical trials; while, many more will enter clinical trials soon. 

In conclusion, dendrimers have a promising future in the pharmaceutical and biotechnology industries. Recent technological advancements have allowed dendrimers to become more sophisticated and readily available. Several dendrimers (a very small number of which are currently undergoing clinical trials using systemic and non-systemic routes of administration) are currently being evaluated in clinical trials, thus providing options to increase their efficacy and reduce toxicity, and facilitating the development of new, active dendrimers. However, designing and synthesizing biodegradable dendritic structures that are soluble in water with appropriate molecular weights, the capability for monodispersion, and pharmacodynamic properties, remains a significant challenge. Dendrimers (such as PAMAM) can be labeled during synthesis by introducing 13-C or even 14-C, or any other suitable agent, at every step of multistep synthesis, to prove the important issue of biocompatibility. The stepwise degradation in model organism can be followed by mass spectrometry (for 13-C-containing low molecular products of degradation) or the use of scintillators (for 14-C). The former method is not harmful to the animal. However, this is a long-term job, and it is necessary to comprehensively evaluate the biocompatibility of dendrimers for their final use (such as PAMAM) as a drug carrier. Due to the complex structure of dendrimers, their complexes or conjugates with ligands or drugs may also require advanced analytical techniques, to accurately characterize the final product. In addition, the scaled-up production process represents another important obstacle for dendrimer-based nanomedicines; this is because dendrimers are usually produced by means of multiple processes. Furthermore, better quality control tests are necessary to ensure that polymers composed of multiple components contain the correct components in the correct proportions. A high degree of reproducibility and stability is necessary for the production of dendrimers when scaling-up production. However, in order to overcome these limitations, biodegradable dendrimers need to be tested and proven following existing clinical testing processes. Recent advances in this field have, therefore, laid the foundation for a wide range of potential medical applications. The advent of nanotechnology, and specifically dendrimer nanoparticles, with their unique suite of characteristics, will facilitate the development of medical products that go beyond a single mode of action, to become multifunctional platforms capable of performing multiple functions, including nanotherapeutics and nanotheranostics.

## Figures and Tables

**Table 1:** Toxicity of dendrimers for different cell types.

Cells	Dendrimers	Dose	Action Time	Toxic Effect	Ref.
Hippocampal neurons	PAMAM G4	0.1, 1, 10, 100 µM	24 h	Disrupt neuron membrane permeability Increase the synaptic vesicle release	[78]
Human bronchial epithelial cells (PBECs) Human lung adenocarcinoma cell (A549)		0.1, 0.5, 1, 5, 10, 50, 100 µM	24, 48 h	NF-κB-dependent cell cycle arrest (only PBECs)	[79]
EA.hy926 endothelial cell line (ATCC CRL-2922)		0.4, 0.8 µM	24 h	Increase endothelial cells elasticity Apoptosis	[80]
Human neural progenitor cells (hNPCs)		0.3, 1, 3, 10 µg/mL	72 h	Inhibit proliferation and neuronal migration	[81]
Chinese hamster fibroblasts (B14) Embryonic mouse hippocampus (mHippoE-18) Rat liver cell (BRL-3A)		10, 40, 80, 120, 200 µM	24 h	ROS and mitochondria mediate apoptosis	[82]
Human glioblastoma cell lines (U87MG, U251MG, U118, and A172)	PAMAM G5	12.5, 25, 50, 100 µg/mL	24 h	Decrease cell viability Autophagy	[83]
Cervical carcinoma HeLa cells (KB cells)		3 µM	2, 24 h	Lysosomal alkalization Mitochondria mediated apoptosis	[84]
Human normal liver cell line (HL7702)		12.5–100 μg/mL	24 h	Induce growth inhibition Damage mitochondria Autophagy	[85]
Human umbilical vein endothelial cells (HUVECs)	PAMAM G2, 7	1–100 µg/mL	24 h	Proinflammatory G1 arrest Apoptosis and necrosis	[75]
Hepatocytes Kupffer cells (KCs)	PAMAM G4.5, 5	1, 2, 4, 10 µM	1, 3, 24, 72 h	Damage hepatocyte function	[86]
HER2-positive breast cancer cell lines (SKBR3 and ZR75) Breast cancer cell lines (MCF10A)	PAMAM G4, 6	0.1–100 µM	24, 48, 72 h	Decrease cell viability Apoptosis	[87]
Human umbilical vein endothelial cells (HUVECs) Human aortal smooth muscle cells (hAoSMCs)	PAMAM G2, 3, 4	0.1–500 mM	24 h	Trigger no tissue factor production of HUVECs Reduce the cell vitality of hAoSMCs	[88]
Human keratinocyte cell (HaCaT) Primary adenocarcinoma cell (SW480)	PAMAM G4, 5, 6	0.01–21.1, 0.03–5.2, 0.01–5.168 µM	24 h	ROS related cell death	[89]
Human lens epithelial cells (HLECs) Retinal pigment epithelium cells (RPEs) Human corneal epithelial cells (HCECs)		5–100 µg/mL	24, 48, 72 h	ROS production Apoptosis	[90]
Mouse macrophage cells		0.08–6 µM 0.03–2 µM 0.013–1 µM	24, 48, 72 h	ROS generation Cytokine secretion Cell death	[91]
Human colon cancer cells (Caco-2)	PAMAM G0-G4	0.1, 1, 10 mM	90, 150, 210 min	Reduce membrane permeability and cell viability	[92]
Platelet	PAMAM G3-G6	1.563–100 µg/mL	15 min	Thrombus	[68]
Normal Leukocyte: peripheral blood mononuclear cells (PBMCs) Acute promyelocytic leukemia Leukocyte (HL-60)		50 µg/mL	5, 24 h	Leukocyte procoagulant activity (PCA)	[93]
A549 cell	PAMAM G3.5, 4.5, 5.5, 7.5 and G1-G8	100 µg/mL	24 h	Autophagy associated with Akt-TSC-mTOR	[94]
Human mesenchymal stem cells (hMSCs)	PAMAM G1.5, 2, 3.5, 4, 5.5, 6	0.5, 10 µg/mL	21 d	Decrease cell survival	[95]
Human melanoma cells (SK-Mel-28) HaCaT cell Caco-2 cell	PAMAM G5 core–PAMAM G2.5 shell tecto-dendrimers	5–50 or 50–150 µM	24 h	Apoptosis	[37]
Human liver hepatocellular carcinoma (HepG2) Human prostate cancer cell lines (DU145)	PAMAM G3.0, 3.5, 4.0, 4.5, 5.0 PPI G3.0, 4.0, 5.0	0.04–600 µM	24 h	Apoptosis	[70]
Breast cancer cell (MCF-7 and MDA-MB-231) Leukemia cell lines (LAMA-84, K562, SD-1, SUP-B15)	PAMAM-derived dendrimers G1 (Phe)_6_, G1 (Dan)_3_, and G2; PPI G3	0.25, 0.5, 1, 5, 10 µM	168 h	Apoptosis Necrosis	[96]
Peripheral blood mononuclear cells (PBMCs)	PPI G4	0.05, 0.5, 5 mg/mL	1 h	DNA strand breaks	[97]
Human hepatoma cell line (HepG2) African green monkey kidney cell line (COS-7)	PPI G5	0.001–1 mg/mL	24, 72 h	Decrease cell viability	[53]
Red blood cells (RBCs)	PPI G0.5-G5.0	1 mg/mL	30 min	Membrane rupture	[4]
Human mononuclear blood cells A549 cells Human gingival fibroblasts (HGFs)	CPD G3, G4	0.01–10 µM	12, 24 h	DNA aggregation Cell cycle arrest Apoptosis	[98]
Human GBM cell lines (T98G and LN229) NB cell line (SH-SY5Y) Rat primary cerebellar neurons (CGC) Normal human astrocytes (NHA)	Peptide dendrimers	0.2, 2, 5, 10, 20 µM	30 min	Affect proliferation and long-term colony formation	[99]
Chinese hamster ovary cells (CHO-K1)	Thiophosphate dendrimer	100 pM; 1, 10, 100 nM; 1, 10 µM	1 h	Increase metabolism No notable toxicity	[100]
Human non-tumorigenic keratinocytes (HaCaT) and squamous carcinoma cells (SCC-15)	Homochiral PAMAM dendrimers	0–300 µM	24 h	No toxicity	[27]
Cricetulus griseus cell lines (B14, BRL 3A, NRK 52E)	Glyco-dendrimers	1–100 µM	24 h	Decreased cell vitality	[77]
Human acute leukemia cell line (HL-60) HaCaT cells Caco-2 cells	Carbosilane ruthenium dendrimers	0.5–5 µM	72 h	Apoptosis	[101]

**Table 2:** Toxicity of dendrimers in different tissues.

Tissue	Dendrimers	Administration Mode	Dose	Action Time	Mechanism	Organ	Toxicity	Ref.
Mice	PAMAM G3.5, 4, 6.5, 7	Intravenous injection	10–1000 mg/kg	10 d	Simulate function of thrombin and plasminogen	Blood	Disseminated intravascular coagulation-like manifestations (DIC)	[62]
	PPI G4 glyco-dendrimers	Intravenous injection	29.25, 58.5, 117 mg/kg and 36.50, 73, 146 mg/kg	14 d	ROS induced autophagy	Nerve	Ataxia Tachypnea and vocalization	[115]
	PAMAM G6	Intraperitoneal injection	1, 5, 10 mg/kg	24 h	Hyperphosphorylation of ERK1/2 and p38 MAPK	Kidney	Affect the signal transduction	[109]
	PAMAM G5	Intraperitoneal injection	100 mg/kg	10 d	Inhibit Akt/mTOR and activate ERK1/2 signaling pathway Autophagy	Liver	Liver weight decrease Hepatocyte necrosis	[85]
	PAMAM G5	Intratracheal injection	7.35, 10.5, 15, 21.43, 30.61 µg/g	48 h	Reduce angiotensin converting enzyme II (ACE 2) activity and imbalance of renin-angiotensin system	Lung	Acute lung injury	[116]
	PAMAM G3, 5.5	Intratracheal administration	50 mg/kg	4, 16 h	Deregulate the Akt-TSC2-mTOR signaling pathway to trigger autophagy			[94]
	PAMAM G6	Intraperitoneal injection	10, 20, 40 mg/kg	4 weeks	Inhibit EGFR signaling	Heart	Impair heart recovery from I/R damage	[117]
	PAMAM G2, 3	Apply	0.3, 3, 6, 30, 300 mg/mL	10 d	High expression of proliferating cell nuclear antigen (PCNA) Abnormal cell proliferation	Skin	Hyperplasia of connective tissue fibers Leukocyte infiltration	[118]
Isolated human jejunum and colonic tissues	PAMAM G3.5, G4	Probe injection	0.1, 1, 10 mM	0–120 min	Interaction with epithelial cell membrane	Gastrointestinal tract	Superficial cortical damage Dose limiting toxicity	[119]
Zebrafish embryo	PAMAM G3.5, 4, 4.5, 5 PPI G3, 4, 5	Treatment	0.2–50 µM	96 h	Membrane instability and apoptosis	Embryo	Increase mortality Reduced heartbeat and blood circulation	[70]
Japanese white rabbits	PAMAM G4, 5, 6	Intravitreal injection	50, 100 µg/mL	3 days or 1 week	Independence on ROS	Eyes	Damage eye function	[90]
